# The Liver Plays a Major Role in Clearance and Destruction of Blood Trypomastigotes in *Trypanosoma cruzi* Chronically Infected Mice

**DOI:** 10.1371/journal.pntd.0000578

**Published:** 2010-01-05

**Authors:** Luiz Roberto Sardinha, Tainá Mosca, Rosa Maria Elias, Rogério Silva do Nascimento, Lígia A. Gonçalves, Daniella Zanetti Bucci, Cláudio Romero Farias Marinho, Carlos Penha-Gonçalves, Maria Regina D'Império Lima, José Maria Álvarez

**Affiliations:** 1 Department of Immunology, Biomedical Sciences Institute, University of São Paulo, São Paulo, São Paulo, Brazil; 2 Instituto Gulbenkian de Ciência, Oeiras, Portugal; 3 Department of Parasitology, Biomedical Sciences Institute, University of São Paulo, São Paulo, São Paulo, Brazil; René Rachou Research Center, Brazil

## Abstract

Intravenous challenge with *Trypanosoma cruzi* can be used to investigate the process and consequences of blood parasite clearance in experimental Chagas disease. One hour after intravenous challenge of chronically infected mice with 5×10^6^ trypomastigotes, the liver constituted a major site of parasite accumulation, as revealed by PCR. Intact parasites and/or parasite remnants were visualized at this time point scattered in the liver parenchyma. Moreover, at this time, many of liver-cleared parasites were viable, as estimated by the frequency of positive cultures, which considerably diminished after 48 h. Following clearance, the number of infiltrating cells in the hepatic tissue notably increased: initially (at 24 h) as diffuse infiltrates affecting the whole parenchyma, and at 48 h, in the form of large focal infiltrates in both the parenchyma and perivascular spaces. Phenotypic characterization of liver-infiltrating cells 24 h after challenge revealed an increase in Mac1^+^, CD8^+^ and CD4^+^ cells, followed by natural killer (NK) cells. As evidence that liver-infiltrating CD4^+^ and CD8^+^ cells were activated, increased frequencies of CD69^+^CD8^+^, CD69^+^CD4^+^ and CD25^+^CD122^+^CD4^+^ cells were observed at 24 and 48 h after challenge, and of CD25^−^CD122^+^CD4^+^ cells at 48 h. The major role of CD4^+^ cells in liver protection was suggested by data showing a very high frequency of interferon (IFN)-γ-producing CD4^+^ cells 24 h after challenge. In contrast, liver CD8^+^ cells produced little IFN-γ, even though they showed an enhanced potential for secreting this cytokine, as revealed by *in vitro* T cell receptor (TCR) stimulation. Confirming the effectiveness of the liver immune response in blood parasite control during the chronic phase of infection, no live parasites were detected in this organ 7 days after challenge.

## Author Summary

Chagas disease, a Latin American illness caused by the protozoan parasite *Trypanosoma cruzi*, has only rare spontaneous cure, and in most patients a small number of parasites persists for life in the blood and tissues, leading to chronic disorders such as cardiomyopathy. In a murine model of chronic *T. cruzi* infection we observed that the liver plays an important role in the clearance of blood-circulating parasites. Moreover, parasite accumulation in this organ is followed by their elimination, an effect that is not immediate but seems to depend on the recruitment of leukocytes and on the local production of IFN-γ, a cytokine known to increase the *T. cruzi*-killing capacity of phagocytes. Our findings contribute to the knowledge of *T. cruzi*-host interaction, showing the participation of a non-lymphoid organ in parasite control. In addition, they contribute to understanding the multifaceted role the liver plays in the immune response.

## Introduction

A main feature of human and murine infections by *Trypanosoma cruzi*, the etiological agent of Chagas disease, is the rarity of spontaneous cure. Despite the generation of a potent anti-parasite immune response, that allows the control of parasitemia at the end of the acute phase, a small number of *T. cruzi* persists in the tissues. From this place, and for the lifetime of the host, the parasites occasionally gain access to the blood, where they can be detected by indirect methods such as xenodiagnosis, hemoculture, subinoculation or PCR [1]–[3].

Non-sterile control of *T. cruzi* at the chronic phase of the infection depends on humoral and cellular mechanisms. Destruction of intracellular amastigotes strongly relies in parasite-specific CD4^+^ and CD8^+^ T cells which act by release of pro-inflammatory cytokines and chemokines and direct cytotoxicity of infected cells [4]–[7]. The clearance of extracellular trypomastigotes is optimized by the coordinated cooperation of antibodies and phagocytes, a process that results in efficient parasite-destruction when phagocytes are primed by inflammatory cytokines, notably by IFNγ [8]. Thus, at the tissues, following rupture of a pseudocyst, released trypomastigotes are opsonized by IgG and subsequently phagocytosed by resident macrophages and recruited monocytes and polymorphonuclear cells [9]. At the blood, clearance of IgG-coated tripomastigotes is supposedly mediated by resident mononuclear phagocytes at the lung, liver and spleen [10]. This process depends on an intact Fc portion of the IgG molecule [11], and although shown to require the participation of C3 complement component, occurs independently of the lytic terminal pathway [12].

Low and continuous release of trypomastigotes to the blood (and tissues) contributes to maintain the high level anti-*T. cruzi* effector activity of chronically-infected mice. Short and long-term effects of this continuous stimulus can be mimicked in an amplified version by intravenous (i.v.) challenge of chronic mice with live trypomastigotes. In this respect, we previously observed that 7–12 days after i.v. challenge of chronic mice with homologous parasites, a booster of the anti-*T. cruzi* effector mechanisms occurs, with increase in anti-*T. cruzi* IgG2a and IgG1 serum antibody levels, intense brief burst in the spleen IFN-γ production, activation of B and T cells and accumulation of class II^+^ non-B cells in the spleen [2]. In this work, continuing our studies on the host-parasite interaction at the chronic phase, we analyzed the short-term effects of an intravenous challenge with trypomastigotes. Parasite clearance was shown to occur to a large extent at the liver, an organ with an efficient resident immunity that responds to the acute *T. cruzi* infection with intense inflammation and high IFN-γ production [13].

## Materials and Methods

### Mice

Six- to 8-week-old female C57Bl/6 mice were bred under specific pathogen-free conditions at the Isogenic Mice Facility, Instituto de Ciências Biomédicas, Universidade de São Paulo, Brazil. Experiments were carried out in accordance to the ethical guidelines for experiments with mice, the protocols being approved by the Health Animal Committee (CEEA) of the University of São Paulo.

### Parasites and infection

*T. cruzi* from the Y strain was maintained by weekly passages in A/J mice. C57Bl/6 mice were infected intraperitoneally (i.p.) with diluted blood containing 1000 trypomastigote forms. Parasitemias were determined by microscopic examination of 5 µl blood samples obtained from the tail vein. Seven to ten months after infection, chronic mice were challenged intravenously (i.v.) with 5×10^6^ tissue culture tripomastigotes of the Y strain obtained from infected LLCMK2 cultures. One hour later, challenged chronic animals or unchallenged chronic controls were sacrificed to estimate the parasite load at the lung, liver and spleen and immunohistochemical analysis of *T. cruzi* at the liver tissue. Moreover, 24 and 48 h after challenge other mice were sacrificed for histological examination and leukocyte population analysis at the liver.

### DNA preparation and real-time PCR for *T. cruzi* quantification

Total DNA from spleen, lung and liver tissues collected from mice at the indicated time points post-infection, were extracted using GenomicPrep Cells and Tissue DNA Isolation kit (Amersham Biosciences), following the manufacturers' protocol. Each real-time PCR reaction contained 40 ng genomic DNA, 0.5 µM of *T. cruzi* 18S rRNA gene (AF303659) - specific primers Tc18S-F 5′- TTGAATTGAGGGCCTCTAAGG-3′ and Tc18S-R 5′- AAAGGTACCACTCCCGTGTTT-3′. The *T. cruzi* quantification reactions were performed according to the manufacturers' instructions on an ABI Prism 7900HT system. The real-time PCR reaction used Applied Biosystems' Power SYBR Green PCR Master Mix. Relative quantification, ΔΔCt method, of specific DNA was normalized for mouse GAPDH gene (GAPDH-F 5′-TGAAGCAGGCATCTGAGGG-3′ and GAPDH-R 5′-CGAAGGTGGAAGAGTGGGAG-3′).

### LIT cultures of hepatic tissue

Live *T. cruzi* parasites in the liver of individual chronic mice were revealed by culture of liver tissue aliquots containing 1.6 or 0.4 mg of tissue homogenate (in quadruplicate), at 28°C, in axenic liver infusion tryptose (LIT) medium. Cultures were screened twice a week, for a month, for epimastigote growth. To avoid contamination of liver samples with peripheral blood, the inferior cava vein was sectioned above the diaphragm and the animals connected to a KDS 200 Two-Syringe Infusion Pump (KD Scientific, New Hope, PA) which delivered sterile phosphate-buffered saline, for 5 min, at a flow rate of 2 ml/min, through the left ventricle.

### Histopathological analysis

Liver tissue specimens were collected and fixed in 10% formalin (Merck, La Jolla, CA) for further processing. Paraffin-embedded tissue sections were stained with hematoxylin-eosin and analyzed by optical microscopy. The hepatic inflammatory infiltrates were photographed using an image analysis system (Image Pro Plus Media Cybernetics, Silver Spring, MD).

### Immunohistochemistry

Silane-coated slides (5 µm) of paraffin-embedded liver tissues from chronic mice or from control or chronic mice that had been inoculated i.v., 1 h before, with 5×10^6^ culture trypomastigotes were dewaxed and hydrated by routine methods before the antigen retrieval procedure. Immunostaining was done by overnight incubation at room temperature with mouse-absorbed chronic immune rat serum anti-Y strain *T. cruzi* parasites. Then, the sections were first incubated for 2 h with biotin-labeled secondary antibody and second with the peroxidase-conjugated biotin-avidin complex (Elite ABC kit, Vector laboratories). Finally, the peroxidase was revealed by immersion in DAB (diaminobenzidine, Sigma). Slides were counterstained with hematoxilin.

### Isolation of intrahepatic leukocytes

Intrahepatic leukocytes were isolated as described [14]. Briefly, after perfusion with phosphate buffer solution (PBS), the liver was removed, a cellular suspension was prepared, treated with collagenase 0.02% (Invitrogen, Carlsbad, CA), washed, admixed with 40% metrizamide (Sigma) solution in PBS and gently overlaid with RPMI 1640 medium supplemented with 1% heat-inactivated fetal calf serum (FCS). Culture medium and supplements were purchased from Invitrogen. After centrifugation at 1500 g and 4°C, leukocytes were harvested from the medium-metrizamide inter-phase.

### Phenotypic characterization of intrahepatic leukocyte populations

The phenotype of intra-hepatic leukocytes was determined using a three-color FACScalibur cytometer (Becton-Dickinson, San José, CA), after staining cells with FITC-, PE-, Cy-chrome- or biotin-conjugated monoclonal antibodies (mAbs) to CD4 (clone H129.19), CD8 (clone 53-6.7), B220 (clone RA3-6B2), CD11b (Mac-1; clone M1/70), NK1.1 (clone PK136), CD69 (clone H1.2F3), CD25 (clone 7D4) and CD122 (clone TMβ1) purchased from PharMingen (San Diego, CA). When using biotin-conjugated mAbs, fluorochrome-labeled streptavidin (PharMingen) was added as a second step reagent. The number of each cell population per liver was determined by multiplying its respective frequency among liver leukocytes by the total number of leukocytes per liver estimated in a Neubauer chamber.

### Intracellular detection of IFN-γ

Intrahepatic leukocytes were cultured overnight with Golgistop at 37°C in a 5% CO_2_ atmosphere, according to the manufacturer's instructions, in the presence or absence of plate-bound anti-CD3 (10 µg/ml; clone 145-2C11) and soluble anti-CD28 (2 µg/ml; clone 37.51) mAbs. After being washed, cells were surface stained with FITC- or Cy-Chrome-conjugated mAbs to CD4, and CD8. Cells were then fixed with the Cytofix/Cytoperm buffer and incubated with PE-labeled mAb to IFN-γ (XMG-1.2) diluted in Perm/Wash buffer. The analysis was done in a FACSCalibur cytometer. All reagents were purchased from PharMingen.

### Statistical analysis

Statistical analysis was performed by ANOVA and Tukey's multiple comparison tests, or unpaired T test, using the GraphPad PRISM 4 software. Differences between two groups were considered significant at p<0.05.

## Results

### The liver is a major site for blood parasite clearance in chronic mice

To study the clearance of bloodstream forms of *T. cruzi*, chronic mice were inoculated i.v. with 5×10^6^ trypomastigotes. Tissue culture forms were used because blood trypomastigotes from 7-day infected mice are coated by IgM antibodies which could interfere in parasite recognition by specific IgG [15].

As previously described [16], parasitaemia is not detected in *T. cruzi*-infected chronic mice by direct microscopic examination. Similarly, when chronic mice were challenged i.v. with 5×10^6^ trypomastigotes we did not detect parasites in the blood any time after challenge. Differently, non-infected mice injected with *T. cruzi* displayed high parasitemias thirty minutes after injection, and small numbers were still be seen after 24 and 48 h (Table 1).

**Table 1:** Parasitaemia in chronically infected mice after challenge with *T. cruzi*.

Time after challenge	*Number of parasites/ml of blood (x10^−4^)*				
	Chronic unchallenged	Chronic challenged	Non-infected challenged		
**30 min**	n.d.	n.d.		66.0±43.7	
**24 h**	n.d.	n.d.		1.2±2.5	
**48 h**	n.d.	n.d.		0.5±0.7	

n.d., not detected.

**Figure 1:**
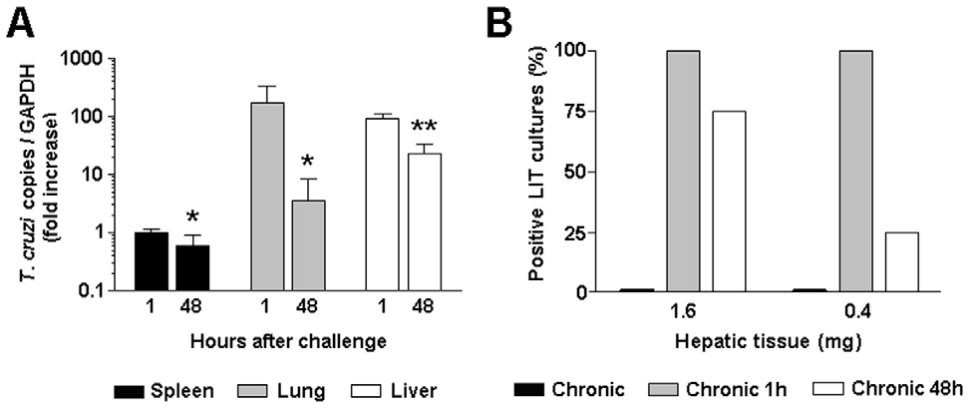
Tissue parasitism in chronic mice challenged with *T. cruzi* trypomastigotes. Chronic mice were analyzed for tissue parasitism 1 h and 48 h after i.v. challenge with 5×10^6^ trypomastigotes. Unchallenged chronic mice were used as controls. (A) Comparison of parasite loads in different tissues using real-time PCR for *T. cruzi* DNA. Average values and standard error for each experimental group (n = 3) are represented. Differences between groups were evaluated by unpaired T test (* p<0.05, ** p<0.0001, compared to 1 h). (B) Presence of live *T. cruzi* parasites in the liver of challenged chronic mice. Parasites in liver tissue fragments of 1.6 and 0.4 mg weight were amplified by LIT culture. Results represent the frequency of *T. cruzi*-positive cultures in each group of mice (quadruplicate cultures per liver; n = 3). The results are representative of two experiments.

In chronic mice, clearance of circulating blood *T. cruzi* parasites is thought to be mediated by resident mononuclear phagocytic cells in the lung, liver and spleen. To assess the relative clearance at these organs, the tissue parasite load of chronic mice was evaluated by real time PCR for *T. cruzi* DNA before and 1 or 48 h after challenge. In unchallenged chronic mice we were unable to amplify *T. cruzi* DNA in any of the three organs (data not shown). Meanwhile, one hour after i.v. challenge of chronic mice, the amounts of *T. cruzi* DNA were sharply increased, notably in the liver and lung and, at lower level, in the spleen (Fig. 1A). Since PCR was done with same amounts of tissue DNA and the weight of the liver was 6.3 times that of the lungs and 12.7 times that of the spleen, we concluded the liver plays a major role in parasite removal. Forty eight hours after challenge the numbers of amplified copies of *T. cruzi* DNA were drastically reduced in these organs. This represented 98% reduction for the lung, 74% reduction for the liver and 39% reduction at the spleen.

To evaluate if parasites removed by the liver were alive, liver fragments obtained 1 and 48 h after challenge of chronic mice were cultured in LIT medium. Liver cultures from unchallenged chronic mice showed no live *T*. cruzi (data not shown). Meanwhile, in 1-h challenged chronic mice, parasites were observed in 100% of cultures containing 0.4 mg of liver tissue, the number of positive cultures being strongly reduced at 48 h of challenge (Fig. 1B). In additional experiments, no parasite was detected in cultures containing 1.6 mg of liver tissue from 7-day challenged chronic mice (data not shown). Differently from liver cultures, no parasite growth was observed by culturing 5 µL aliquots of blood from chronic mice, before, or 1 and 48 h after challenge, in contrast with blood cultures from challenged control mice that yielded 100% positivity (data not shown).

**Figure 2:**
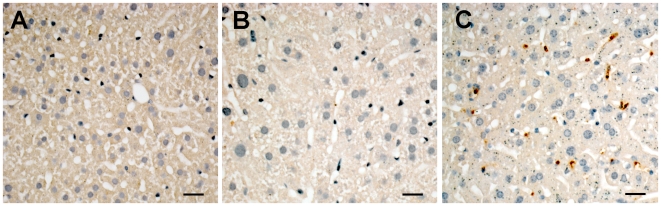
Presence of *T. cruzi* parasites at the liver parenchyma of chronic mice challenged with trypomastigotes. *T. cruzi*-infected chronic mice and non-infected controls were injected i.v. with 5×10^6^ culture trypomastigotes and 1 h later, the animals were sacrificed and the liver tissue screened for the presence of parasite antigen by immunohistochemistry as described in material and methods. Unchallenged chronic mice were included as controls. Pictures are representative of three mice in each group. Challenged control mice (A); unchallenged chronic mice (B); challenged chronic mice (C). Bar: 20 µm.

To visualize the parasites at the liver parenchyma of challenged chronic mice, the liver tissue was analyzed by immunohistochemistry using a mouse-absorbed rat antiserum specific for *T. cruzi* Y parasites. As shown in figure 2, intact or damaged parasites were seen uniformly distributed along the liver parenchyma of 1-h challenged chronic mice. Contrarily, in challenged control mice and in unchallenged chronic mice, peroxidase-positive staining was not observed.

### Analysis of intra-hepatic leukocytes in challenged chronic mice

**Figure 3:**
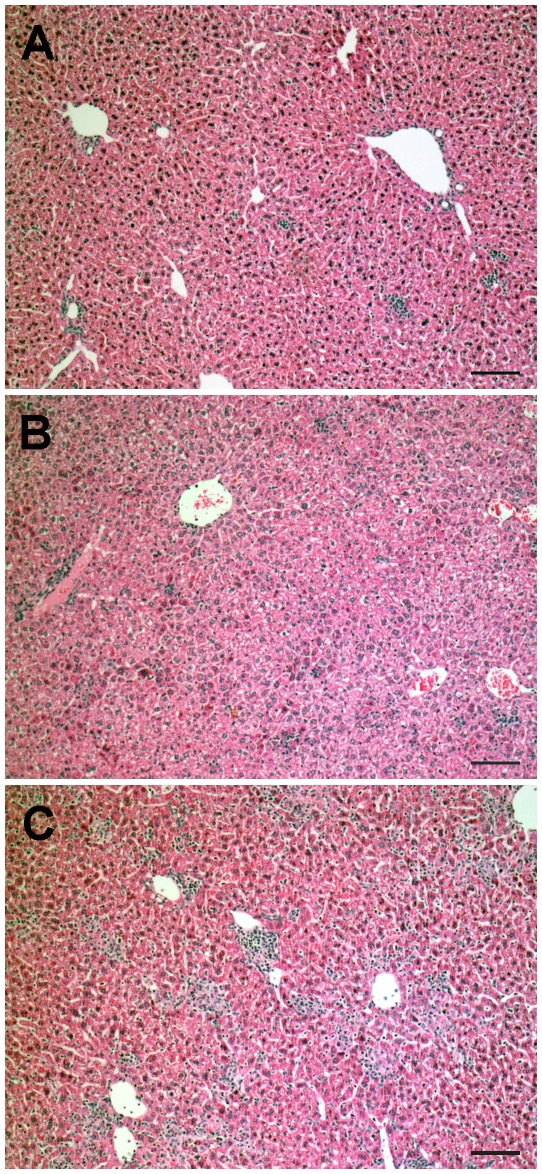
Histopathological analysis of liver in unchallenged and challenged chronic mice. C57BL/6 mice were infected with *T. cruz*i and after 8 months challenged i.v. with 5×10^6^ homologous culture trypomastigotes. Chronic mice (A) and chronic mice, 24 h (B) or 48 h (C) after challenge. Lesions are representative of at least 6–10 animals in each group. Bar: 100 µm.

Histological examination of the liver tissue in chronic mice revealed a small number of focal infiltrates in the parenchyma and few perivascular infiltrates of discrete intensity (Figure 3A). Twenty four hours after challenge, a moderate increase in the number of parenchyma-scattered leukocytes, many of which grouped as tiny foci of 5–10 cells, was observed (Figure 3B). At 48 h, the above picture progressed to harbor a high number of parenchyma-scattered leukocytes and large-sized focal and perivascular infiltrates (Figure 3C). Viable or damaged amastigote nests were not visualized in the liver of chronic mice or challenged chronic mice, in spite that they were exhaustively sought after. In contrast to the liver, tissue pathology at the heart or striated muscle (quadriceps) was not modified after challenge of chronic mice (data not shown).

**Figure 4:**
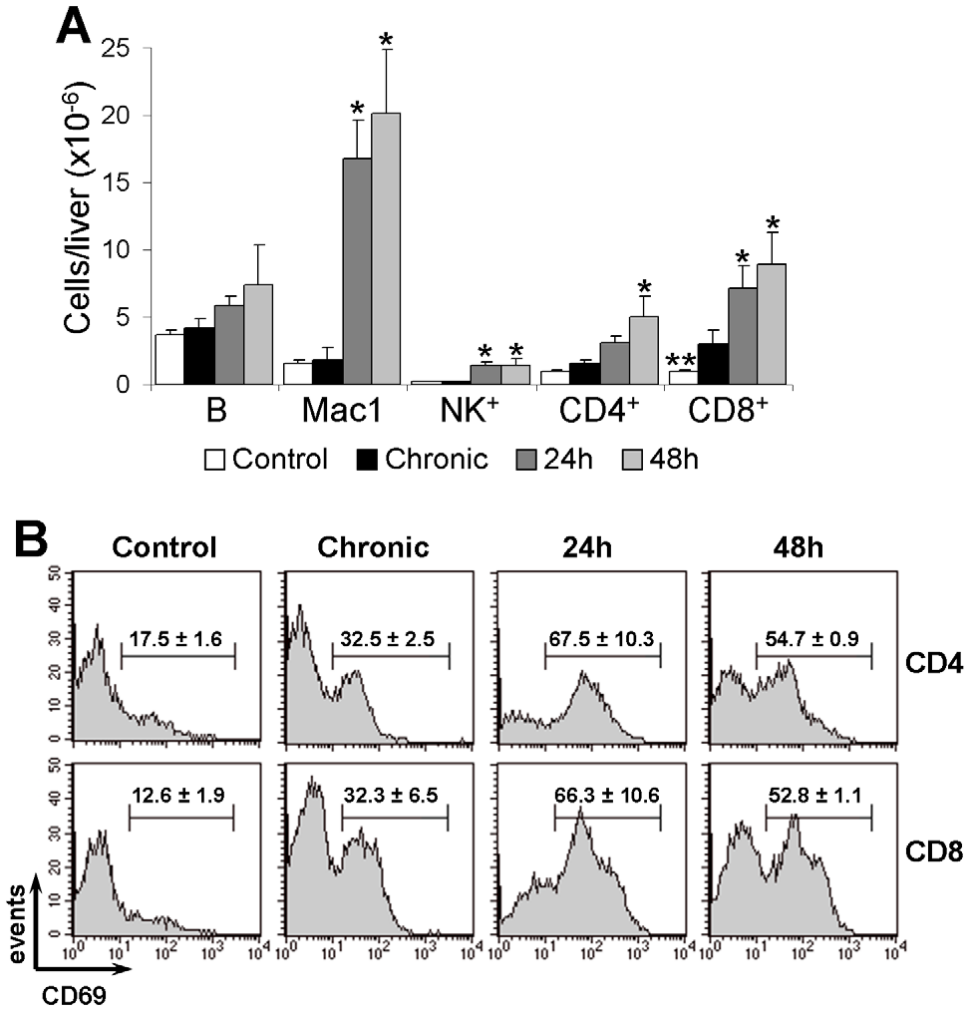
Phenotypic characterization of cellular infiltrates in the liver of unchallenged and challenged chronic mice. C57BL/6 mice infected for 7–10 months with *T. cruzi* parasites were challenged i.v. with 5×10^6^ homologous trypomastigotes and, after 24 and 48 h, liver leukocytes analyzed by flow cytometry. Control and chronic mice were also included. (A) Mean±SD (n = 3) of total liver numbers of B (B220^+^), Mac1^+^ (Mac1^+^CD4^−^CD8^−^B220^−^), NK (NK1.1^+^CD4^−^CD8^−^), CD4^+^ and CD8^+^ cells; Differences between groups were evaluated by ANOVA and Tukey's multiple comparison tests (* p<0.05 and ** p<0.005, compared to chronic mice). A representative experiment out of three is shown. (B) CD69 expression by gated CD4^+^ and CD8^+^ cells. Numbers indicate the mean±SD (n = 3) of the percentage of CD69^+^ cells. Representative histograms of one experiment out of two are shown.

To analyze the cellular composition of liver infiltrates, intra-hepatic leukocytes from chronic mice, challenged chronic mice and controls were characterized by flow cytometry. Total number of intra-hepatic leukocytes were discretely increased in chronic mice compared to control mice (11.7±3.6×10^6^ cells versus 7.4±0.4×10^6^ cells; p<0.01). At the chronic phase, B (B220^+^) cells were the most numerous population, but showed no changes in relation to control mice (Figure 4). Besides, no significant changes were observed for Mac1^+^ (Mac1^+^CD4^−^CD8^−^B220^−^) cells and NK (NK1.1^+^CD4^−^CD8^−^) cells. CD8^+^ and CD4^+^ cells were significantly increased in relation to control mice, with predominance of CD8^+^ cells. In consonance with the histological analysis, a huge increase in intra-hepatic leukocytes occurred in challenged mice (from 11.7±3.6×10^6^ cells in chronic mice to 35.8±1.1×10^6^ and 43.4±11.9×10^6^ cells at 24 and 48 h after challenge, respectively). After challenge, Mac1^+^ cells became the most frequent liver leukocyte population, representing the highest cell increase in relation to chronic mice. CD8^+^ and CD4^+^ cells progressively increased after challenge, the CD4/CD8 ratio being maintained as in chronic mice. In additional experiments it was observed, for both unchallenged and challenged chronic mice, that the Mac1^+^ liver population included GR-1^LOW^ and GR-1^HIGH^ cells [17], where GR-1^HIGH^ cells accounted for around one third of Mac1^+^ cells, 48 h after challenge (Supplementary Figure S1).

The activation status of intra-hepatic CD4^+^ and CD8^+^ cells was estimated by expression of CD69, an early lymphocyte activation marker [18]. For both T cell subsets, CD69 expression was higher in chronic mice than in control mice (Figure 4). However, when chronic mice were challenged with trypomastigotes the frequency of CD4^+^CD69^+^ and CD8^+^CD69^+^ cells drastically increased after 24 h, slightly declining at 48 h. Challenge-induced augments in CD69 expression correlated with increases in the frequencies of large cells. Thus, for CD4^+^ cells, the percentage of large cells increased from 23% in chronic mice to around 60% at 24 and 48 h of challenge. For CD8^+^ cells, a notable increase was also found, from 30% in chronic mice to 55% and 64% at 24 and 48 h of challenge, respectively (Supplementary Figure S2).

### Expression of the IL-2 receptor α and β chains by intra-hepatic CD4^+^ cells in challenged chronic mice

To further evaluate the activation status of intra-hepatic CD4^+^ cells we investigated the surface expression of CD25 and CD122 molecules. The CD25 molecule constitutes the IL-2 receptor (IL-2R) α chain, which structures the high affinity IL-2R when associated to the β (CD122) and cγ (CD132) chains, and the low affinity IL-2R, when associated to just the cγ chain [19]. In addition, the IL-2R β chain is also used, together with the cγ and the IL-15R α chains, to structure in the IL-15R [20]. In mice, regulatory T cells usually have a CD25^+^CD122^LOW^ phenotype, while activated/effector T cells are CD25^+^CD122^HIGH^ and memory cells display a CD25^−^CD122^HIGH^ phenotype [21].

**Figure 5:**
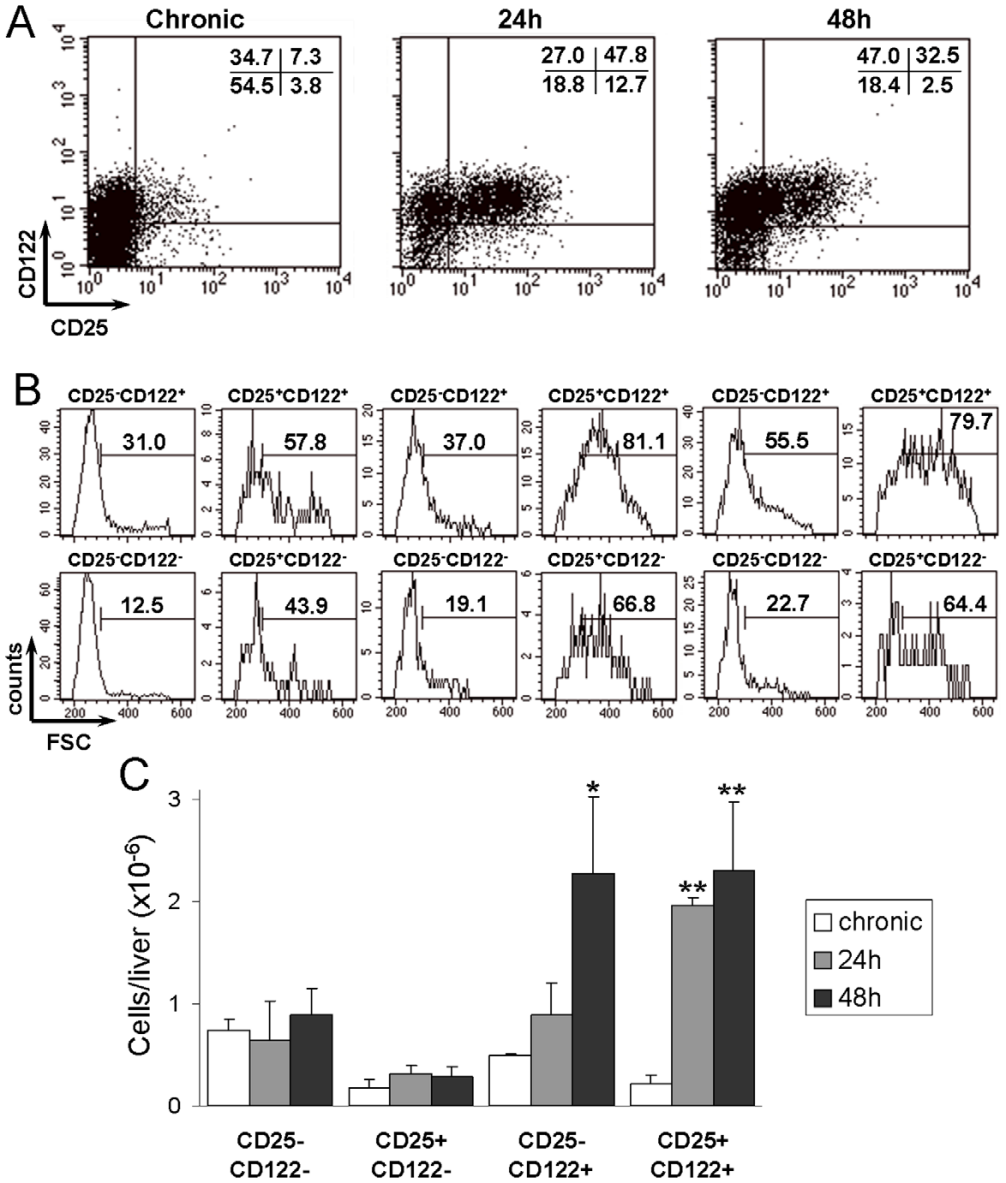
Expression of CD25 and CD122 by CD4^+^ cells in the liver of chronic mice challenged with *T. cruzi* trypomastigotes. C57BL/6 mice infected for 7 months with *T. cruzi* were challenged i.v. with 5×10^6^ trypomastigotes and, after 24 and 48 h, the liver leukocytes analyzed by flow cytometry. (A) Expression of CD25 and CD122 by CD4^+^ cells. Frequency of large cells (B) and total liver cell numbers (C) of CD4^+^ subpopulations defined by expression of CD25 and CD122 molecules. Numbers in dot blots indicate the mean frequencies of cells in quadrants. Numbers in histograms indicate the mean frequencies of large cells among cell subsets defined by quadrants in A. Data in C correspond to the mean±SD of total liver cell numbers of the indicated subpopulations. A representative experiment (n = 3) of two is shown. Differences between groups were evaluated by ANOVA and Tukey's multiple comparison tests (* p<0.01; ** p<0.001, compared to chronic mice).

In chronic mice, small fractions of liver CD4^+^ cells bore the CD25^+^CD122^+^ and CD25^+^CD122^LOW/NULL^ phenotypes, while around 30% had a CD25^−^CD122^+^ phenotype (Figure 5A). Twenty-four hours after challenge, half the CD4^+^ cells showed a CD25^+^CD122^+^ phenotype, the expression of the CD25 molecule in this cell subset being notably higher than that of corresponding cells in chronic mice. Nonetheless, after 48 h, the frequency of CD25^+^CD122^+^CD4^+^ cells diminished to 30%, their level of CD25 expression being also reduced. Conversely, the frequency of CD25^−^CD122^+^CD4^+^ cells decreased 24 h after challenge, increasing again at 48 h.

CD25^+^CD122^+^CD4^+^ cells in the liver of challenged mice were predominantly blasts, both at 24 and 48 h. For the CD25^−^CD122^+^CD4^+^ cell subset, however, the frequency of large cells in 24-h challenged chronic mice was considerably lower, similar to that in chronic mice (around 30–40%), increasing to 55% by 48 h (Figure 5B).

When these data were expressed in terms of total cell numbers in the liver, we observed that CD25^+^CD122^+^CD4^+^ cells notably increased 24 h after challenge, the augment being maintained at 48 h. Differently, recruitment/differentiation of CD25^−^CD122^+^CD4^+^ cells was relatively delayed, a significant increase being only observed after 48 h (Figure 5C).

### IFN-γ production by CD4^+^ and CD8^+^ liver cells after challenge of chronic mice with tripomastigotes

IFN-γ plays a crucial role in *T cruzi* infection, acting at various levels, the optimization of macrophage trypanocidal activity being decisive [22]. During the acute phase of experimental Chagas disease different cell types produce IFN-γ in the liver, contributing to parasite clearance at this organ [13]. At the chronic phase of *T. cruzi* infection, however, dysfunctional CD8^+^ cells with impaired effector functions, inclusive deficient IFN-γ production, have been described in non-lymphoid tissues [23]. To evaluate if the intra-hepatic CD4^+^ and CD8^+^ T cell subsets produced IFN-γ after trypomastigote challenge, we examined the *ex vivo* production of this cytokine, by intracellular staining, in liver cells of chronic mice, before and 24 or 48 h after challenge with *T. cruzi*.

**Figure 6:**
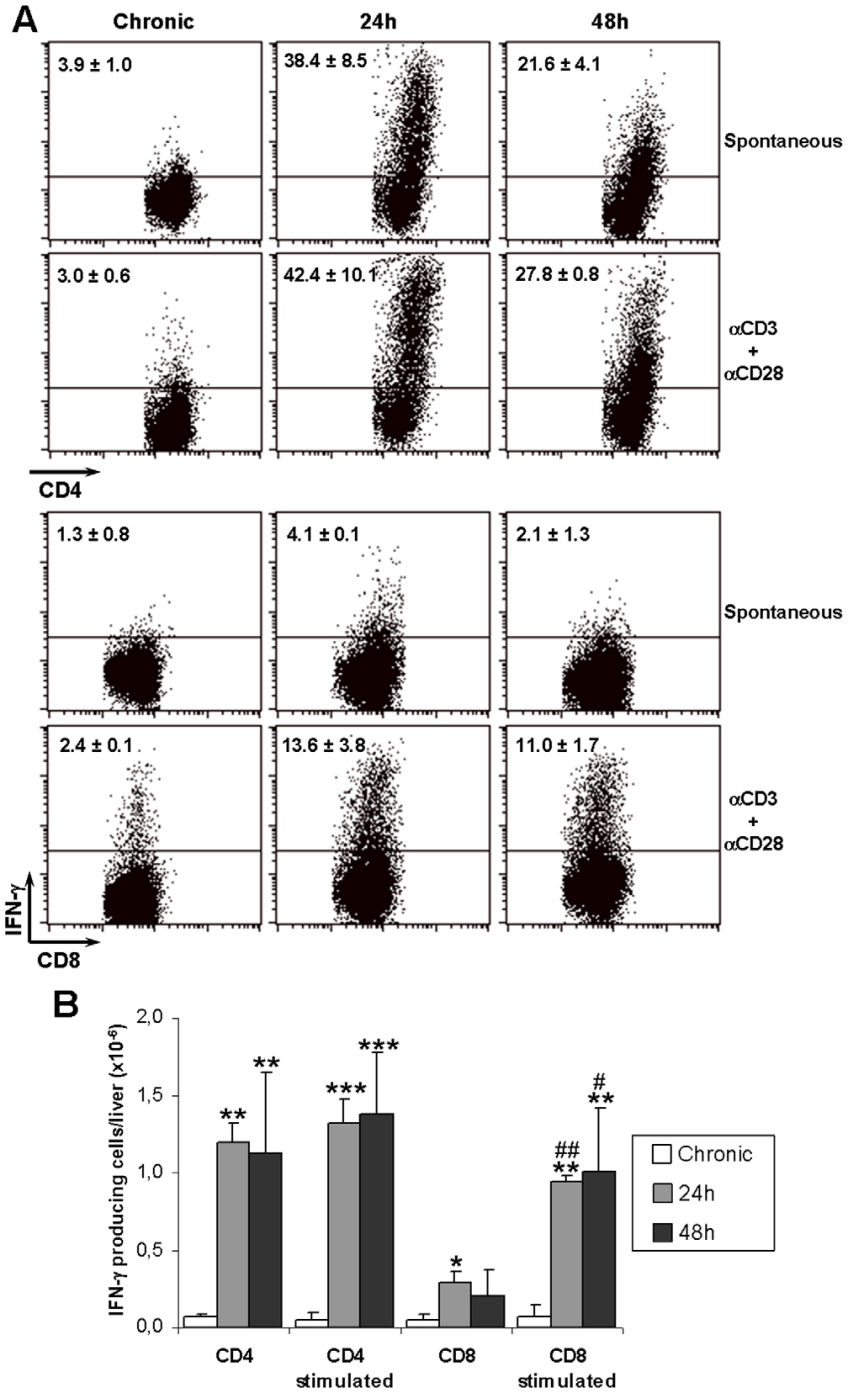
IFN-γ production by CD4^+^ and CD8^+^ liver cells after challenge of chronic mice with *T. cruzi* tripomastigotes. Frequency (A) and total numbers (B) of IFNγ-producing cells in gated CD4^+^ and CD8^+^ cells of unchallenged and *T. cruzi*-challenged chronic mice. The spontaneous and anti-CD3/CD28-stimulated production of IFNγ was evaluated 24 and 48 h after challenge. Numbers inside dot-blots represent the mean±SD (n = 3) of IFNγ-producing cell frequencies. Bars in (B) represent the mean±SD (n = 3) of IFNγ-producing cell numbers per liver of mice in (A); * p<0.05; ** p<0.01; *** p<0.001 (compared to chronic mice by ANOVA and Tukey's multiple comparison tests). # p<0.05; ## p<0.0001 (compared to spontaneous production by unpaired T test). A representative experiment out of two is shown.

CD4^+^ and CD8^+^ liver cells from chronic mice displayed low frequencies of IFN-γ producing cells, which were not significantly modified after *in vitro* stimulation with anti-CD3/CD28 mAbs (Figure 6A–B). Twenty-four hours after challenge, however, a huge increase in the frequency of CD4^+^ cells producing IFN-γ was observed. This frequency slightly decreased at 48 h of challenge, but, because of the augment in total CD4^+^ cell numbers, the population of IFN-γ producing CD4^+^ cells in the liver remained of the same size at 24 h and 48 h (Figure 6B). For these cells, *in vitro* stimulation with anti-CD3/CD28 mAbs did not resulted in additional increase in the frequency of IFN-γ-producing cells. Differently from CD4^+^ cells, challenge of chronic mice resulted in discrete increases in the frequency (Figure 6A) and total liver number (Figure 6B) of IFN-γ-producing CD8^+^ cells. However, after *in vitro* stimulation with anti-CD3/CD28 mAbs, important increases in these values were observed.

## Discussion

In chronic host tissues, trypomastigotes released upon rupture of an amastigote nest immediately bind specific IgG facilitating their internalization by resident or recruited phagocytes [24], a process where the production of nitric oxide and oxygen radicals by phagocytes results in destruction of the ingested parasites [22]. Yet, the uptake of opsonized trypomastigotes by tissue phagocytes is not totally effective as a fraction of locally-released parasites manage to reinvade neighboring cells or make their way to the intravascular space. Once inside blood vessels, IgG-coated trypomastigotes are quickly cleared at the lung, liver and spleen by a mechanism that depends on the Fc portion of the IgG, but not on the lytic complement pathway [25]. This was elegantly shown by Umekita and Mota [11] by observing that trypomastigotes injected i.v. into normal mice are rapidly cleared from the blood following i.v. administration of immune mouse serum or parasite-specific IgG.

In this paper, by demonstrating high levels of *T. cruzi* DNA in the liver of challenged chronic mice we conclude this organ plays a major role for the removal of blood circulating parasites in chronic mice. Meanwhile, because trypomastigotes disappear from the blood of chronic mice soon after i.v. inoculation, but live parasites are still found in the liver after 48 h, we conclude the uptake of IgG-opsonized *T. cruzi* by liver phagocytes is not immediately followed by their destruction. Failure to rapidly destroy the opsonized parasites was an unexpected finding considering the extensively documented synergism of IFN-γ and specific IgG for macrophage killing of *T. cruzi* [8],[24]. Since serum from chronic C57BL/6 mice contained high levels of anti-*T. cruzi* IgG antibodies (data not shown), this failure could have occurred because of insufficient signaling of resident liver phagocytes (Kuppfer cells) by systemic or locally-released IFN-γ or other macrophage-activating cytokines, or because of an intrinsic resistance to priming of liver phagocytes. Independently of the reasons behind, our results indicate that, in chronic mice, resident liver phagocytes are not fully-optimized in their trypanocidal activity for IgG-coated parasites.

Meanwhile, the liver trypanocidal activity seems to be increased after recruitment of lymphocytes that produce IFN-γ. This is suggested by the absence of amastigote nests at the liver in the days following challenge and by the absence of positive LIT cultures in liver samples obtained from 7-day challenged chronic mice. Yet, the possibility that *T. cruzi* is not destroyed at the liver, the parasitized cells leaving this organ through the bloodstream to be destroyed elsewhere, is remote due to the fact that LIT cultures in blood samples were also negative. Our observation that intra-hepatic CD4^+^ cells produced high levels of IFN-γ 24 h after clearance indicates that following blood parasite clearance the liver becomes an important source of this cytokine. Moreover, because IFN-γ production was induced in CD4^+^ cells, but only marginally in CD8^+^ cells, it is possible that following immune clearance successful presentation of parasite antigens was predominantly achieved through MHC class II molecules. This interpretation would imply that most cleared parasites are retained in the phagocytic vacuole of liver phagocytes with few of them escaping to the cytosol or invading hepatocytes, even after 48 h. Alternatively, the low spontaneous production of IFNγ by CD8^+^ cells could be explained by their commitment to cytolytic function rather than cytokine production. Interestingly, many of the CD8^+^ cells in the liver of challenged chronic mice were blasts that expressed the activation marker CD69, and indication they had been signaled and might not be totally quiescent. It remains to be determined whether CD8^+^ cell activation had occurred through class I-peptide recognition or cytokines.

In addition to IFN-γ production, CD4^+^ cells in the liver of challenged chronic mice presented other signs of activation, two thirds of the cells expressing the early activation marker CD69 [18]. Moreover half the CD4^+^ cells were large CD25^+^CD122^+^ cells, a phenotype of effector cells. Besides this population, the liver of challenged mice contained CD25^−^CD122^+^CD4^+^ cells, a population with reduced number of blasts, which could be memory cells that respond to IL-15 [26]. The fact that the liver of unchallenged chronic mice contains a large number of CD25^−^CD122^+^CD4^+^ cells, but few CD25^+^CD122^+^CD4^+^ cells, together with our observation that the level of CD25 expression in CD25^+^CD122^+^CD4^+^ cells decreases after 48 h of challenge, raises the possibility that at least part of the CD25^+^CD122^+^CD4^+^ population differentiates *in situ* to CD25^−^CD122^+^CD4^+^ memory cells.

In spite that the IFN-γ production response to clearance seemed to be CD4^+^ cell-mediated, CD8^+^ cells predominated in the liver of challenged chronic mice. Diverse groups have reported, in humans and mice, that CD8^+^ cells constitute the predominant lymphocyte subset in inflammatory sites of *T. cruzi*-infected chronic hosts [16], [27]–[30]. Moreover, a large fraction of CD8^+^ cells in chronic mice display an effector memory (EM) phenotype [30]–[32], inasmuch as they do not spontaneously produce IFN-γ and other inflammatory cytokines, but they can do so after engaging *T. cruzi*-infected targets [30]. In consequence, CD8^+^ cells expanded/recruited to the liver tissue of challenged chronic mice seem to be EM cells and not anergic or dysfunctional [23], since they could be induced to produce IFN-γ upon *in vitro* stimulation with anti-CD3/CD28 mAbs. By expanding/recruiting a large number of CD8^+^ EM cells, the liver of the chronic host is prepared for the eventual colonization of non-phagocytes by trypomastigotes, a process that seems to be of little magnitude.

From the experiments shown in this work we can extrapolate that by clearing a large fraction of blood trypomastigotes and recruitment of IFNγ-producing effector T lymphocytes the liver plays an important role in the control of low level parasitaemia of chronic mice. It should be considered, however, that *T. cruzi* parasites are extremely diverse, exhibiting differences, not only in relation to tropism, but also in resistance to the immune effector mechanisms [33],[34]. Therefore, because our data derive from experiments with Y strain parasites, a prototypic rethyculotropic strain, additional experiments will be necessary to ascertain the extent to which these conclusions are valid for other *T. cruzi* parasites.

## Supporting Information

Figure S1 GR-1 expression by Mac-1^+^CD4^−^CD8^−^B220^−^ cells in the liver of unchallenged and challenged chronic mice.
(0.01 MB PDF)

Figure S2 Frequency of large cells among CD4^+^ and CD8^+^ cells in the liver of unchallenged and challenged chronic mice.
(0.01 MB PDF)
