# Supplementary material for: The Liver Plays a Major Role in Clearance and Destruction of Blood Trypomastigotes in Trypanosoma cruzi Chronically Infected Mice
Source: PLoS Negl Trop Dis. 2010 Jan 5;4(1):e578. doi: 10.1371/journal.pntd.0000578 (PMC2793026; doi:10.1371/journal.pntd.0000578)
Supplement: Figure S1 — GR-1 expression by Mac-1+CD4−CD8−B220− cells in the liver of unchallenged and challenged chronic mice. (0.01 MB PDF) [file pntd.0000578.s001.pdf]

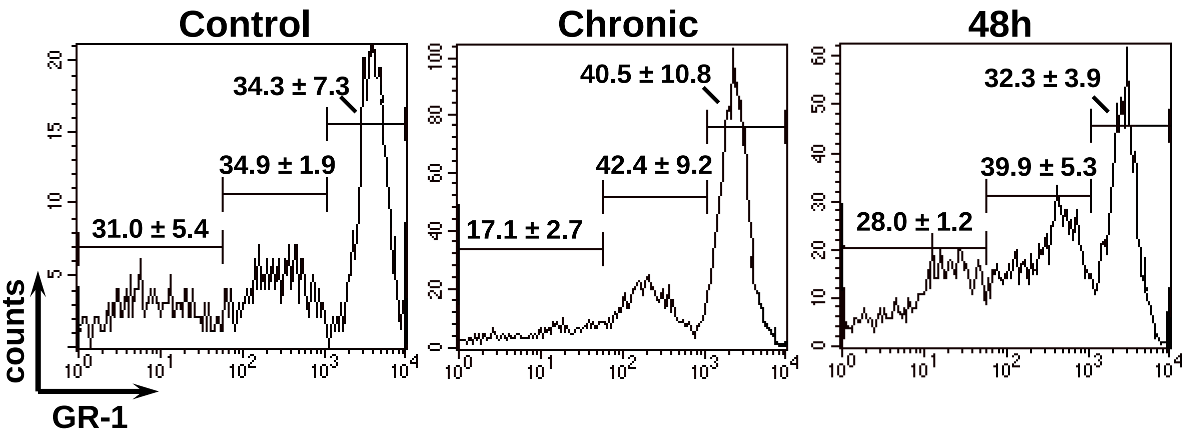

Supplementary figure S1 – GR-1 expression by Mac-1^{+}CD4^{-}CD8^{-}B220^{-} cells in the liver of unchallenged and challenged chronic mice. C57BL/6 mice infected for 7 months with *T. cruzi* parasites were challenged i.v. with 5x10^{6} homologous trypomastigotes and, after 48 h, the liver leukocytes analyzed by flow cytometry. Control and chronic mice were also included. GR-1 expression by gated Mac1^{+}CD4^{-}CD8^{-}B220^{-}. Numbers indicate the mean ± SD of percentages of cells with low, intermediate and high GR-1 expression. Representative histograms of one experiment (n=3) out of two are shown.
