# Supplementary material for: The Liver Plays a Major Role in Clearance and Destruction of Blood Trypomastigotes in Trypanosoma cruzi Chronically Infected Mice
Source: PLoS Negl Trop Dis. 2010 Jan 5;4(1):e578. doi: 10.1371/journal.pntd.0000578 (PMC2793026; doi:10.1371/journal.pntd.0000578)
Supplement: Figure S2 — Frequency of large cells among CD4+ and CD8+ cells in the liver of unchallenged and challenged chronic mice. (0.01 MB PDF) [file pntd.0000578.s002.pdf]

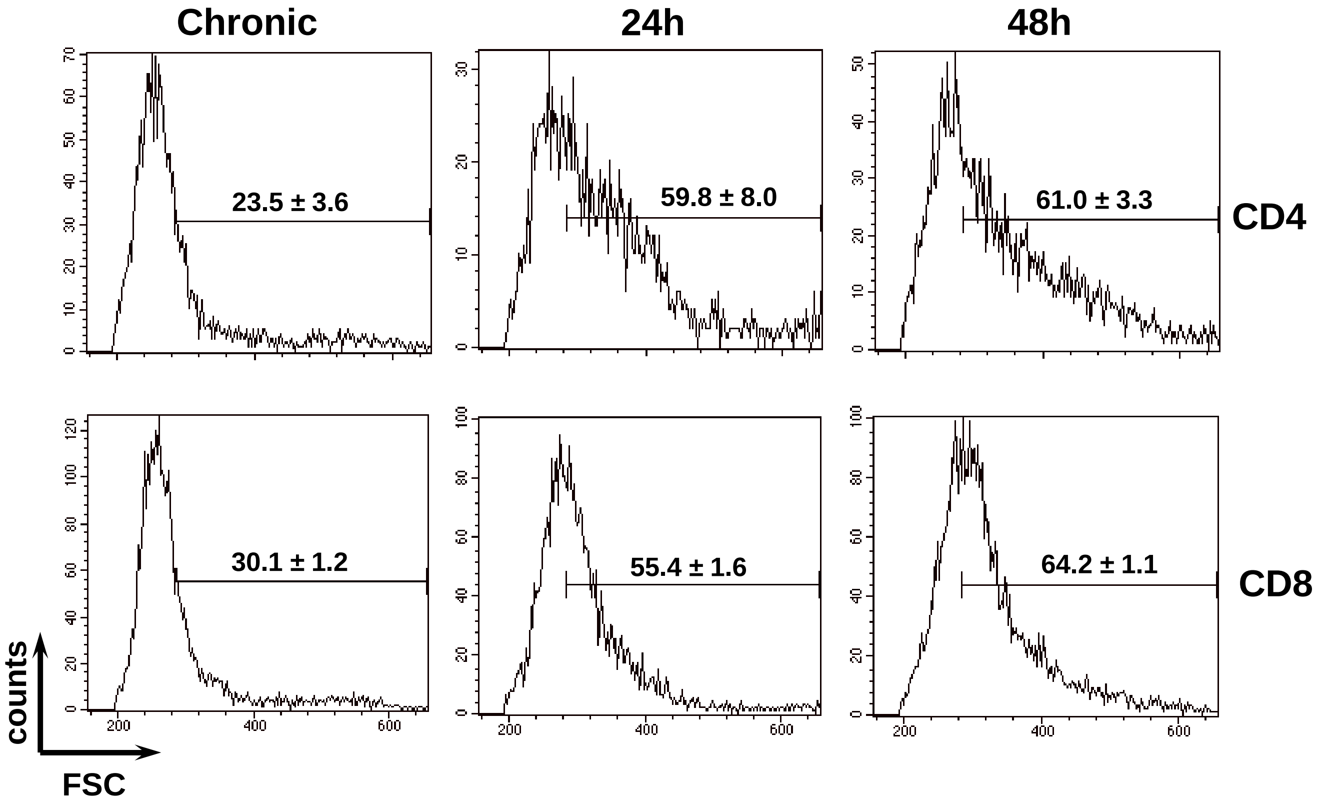

Supplementary figure S2 – Frequency of large cells among CD4$^+$ and CD8$^+$ cells in the liver of unchallenged and challenged chronic mice. C57BL/6 mice infected for 7 months with *T. cruzi* parasites were challenged i.v. with $5x10^6$ homologous trypomastigotes and, after 24 and 48 h, the liver leukocytes analyzed by flow cytometry. Frequency of large cells in gated CD4$^+$ cells (upper) and gated CD8$^+$ cells (lower). Numbers in histograms indicate the mean frequencies of large cells in each subset. Representative histograms of one experiment (n=3) out of two are shown.
